# 1-(5-Bromo-1-benzofuran-2-yl)ethanone

**DOI:** 10.1107/S160053681201985X

**Published:** 2012-05-12

**Authors:** Hoong-Kun Fun, Ching Kheng Quah, Hatem A. Abdel-Aziz

**Affiliations:** a X-ray Crystallography Unit, School of Physics, Universiti Sains Malaysia, 11800 USM, Penang, Malaysia; b Department of Pharmaceutical Chemistry, College of Pharmacy, King Saud University, PO Box 2457, Riyadh 11451, Saudi Arabia

## Abstract

The title compound, C_10_H_7_BrO_2_, is approximately planar (r.m.s. deviation = 0.057 Å for the 13 non-H atoms). In the crystal, mol­ecules are linked *via* C—H⋯O hydrogen bonds into *C*(5) chains propagating in [100].

## Related literature

For general background to and the biological activity of benzofuran derivatives, see: Abdel-Aziz *et al.* (2009 ▶); Abdel-Aziz & Mekawey (2009 ▶); Bhovi *et al.* (2009 ▶); Abdel-Wahab *et al.* (2009 ▶); Csaba *et al.* (2003 ▶); Bevinakatti & Badiger (1982 ▶). For reference bond lengths, see: Allen *et al.* (1987 ▶). For the stability of the temperature controller used in the data collection, see Cosier & Glazer (1986 ▶).
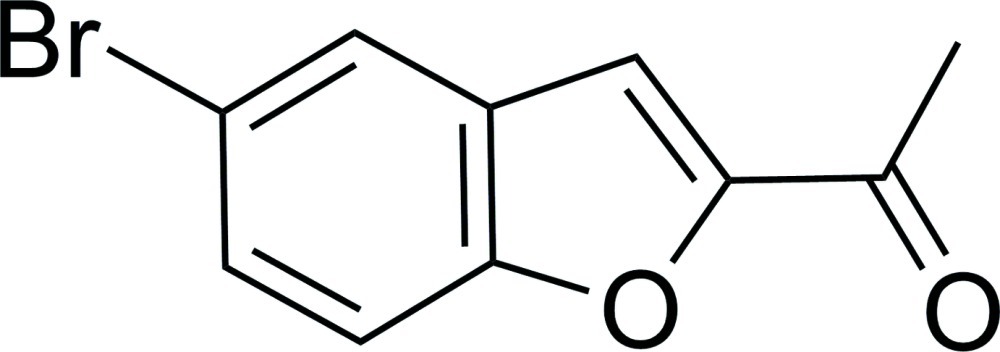


## Experimental

### 

#### Crystal data

C_10_H_7_BrO_2_
*M* *_r_* = 239.07
Orthorhombic, 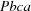

*a* = 10.8301 (2) Å
*b* = 7.4630 (1) Å
*c* = 21.7213 (3) Å
*V* = 1755.62 (5) Å^3^
*Z* = 8
Mo *K*α radiation
μ = 4.64 mm^−1^
*T* = 100 K
0.26 × 0.19 × 0.18 mm

#### Data collection

Bruker SMART APEXII DUO CCD diffractometer
Absorption correction: multi-scan (*SADABS*; Bruker, 2009 ▶) *T*_min_ = 0.377, *T*_max_ = 0.482
43601 measured reflections
3331 independent reflections
2689 reflections with *I* > 2σ(*I*)
*R*_int_ = 0.038

#### Refinement

*R*[*F* ^2^ > 2σ(*F* ^2^)] = 0.024
*wR*(*F* ^2^) = 0.058
*S* = 1.04
3331 reflections
119 parameters
H-atom parameters constrained
Δρ_max_ = 0.47 e Å^−3^
Δρ_min_ = −0.28 e Å^−3^

### 

Data collection: *APEX2* (Bruker, 2009 ▶); cell refinement: *SAINT* (Bruker, 2009 ▶); data reduction: *SAINT*; program(s) used to solve structure: *SHELXTL* (Sheldrick, 2008 ▶); program(s) used to refine structure: *SHELXTL*; molecular graphics: *SHELXTL*; software used to prepare material for publication: *SHELXTL* and *PLATON* (Spek, 2009 ▶).

## Supplementary Material

Crystal structure: contains datablock(s) global, I. DOI: 10.1107/S160053681201985X/hb6772sup1.cif

Structure factors: contains datablock(s) I. DOI: 10.1107/S160053681201985X/hb6772Isup2.hkl

Supplementary material file. DOI: 10.1107/S160053681201985X/hb6772Isup3.cml

Additional supplementary materials:  crystallographic information; 3D view; checkCIF report

## Figures and Tables

**Table 1:** Hydrogen-bond geometry (Å, °)

*D*—H⋯*A*	*D*—H	H⋯*A*	*D*⋯*A*	*D*—H⋯*A*
C7—H7*A*⋯O2^i^	0.95	2.45	3.3495 (16)	158

Symmetry code: (i) 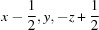.

**Fig. 1.**
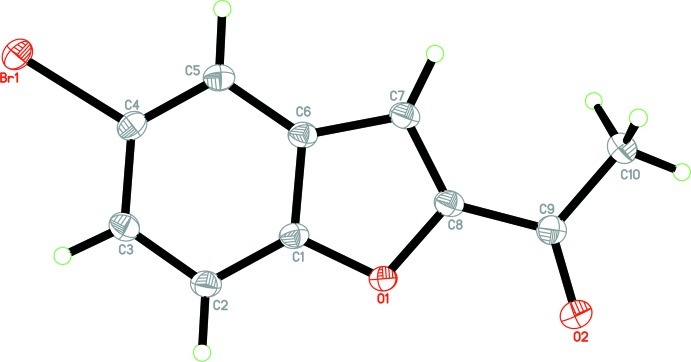
The molecular structure of the title compound showing 50% probability displacement ellipsoids for non-H atoms.

**Fig. 2.**
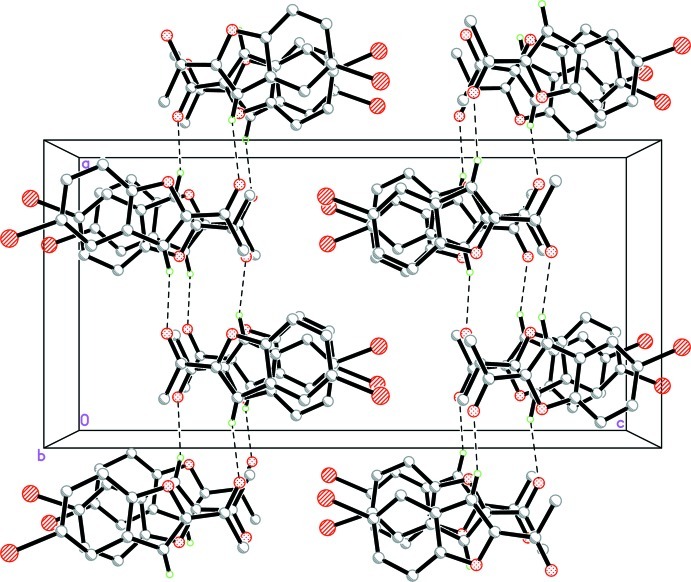
The crystal structure of the title compound, viewed along the b axis. H atoms not involved in hydrogen bonds (dashed lines) have been omitted for clarity.

## supplementary crystallographic information

### Comment

Benzofuran derivatives have useful biological activities, such as anticonvulsant, anti-infammatory, antitumor and antihistaminic activities. They were also found to be useful as antifungal, anthelmintic and antihyper-glycemic agents (Abdel-Aziz *et al.*, 2009; Abdel-Aziz & Mekawey, 2009; Bhovi *et al.*, 2009). Due to their considerable biological activities and in continuation of our interests in the chemistry and biological activities of benzofurans (Abdel-Aziz *et al.*, 2009; Abdel-Aziz & Mekawey, 2009; Abdel-Wahab *et al.*, 2009), the title compound (I) was synthesized to study the structure activity relationships with other benzofurans.

The title compound, Fig. 1, is approximately planar (r.m.s. deviation = 0.057 Å for the 13 non-H atoms). Bond lengths (Allen *et al.*, 1987) and angles are within normal ranges.

In the crystal (Fig.2), molecules are linked *via* C7–H7A···O2 hydrogen bonds (Table 1) into chains propagating in [100].

### Experimental

The title compound was prepared by heating of 5-bromo-salicylaldehyde with chloroacetone in the presence of potassium hydroxide in methanol for 2 h (Csaba *et al.*, 2003; Bevinakatti & Badiger, 1982). Colourless blocks were obtained by slow evaporation from EtOH/DMF solution.

### Refinement

All H atoms were positioned geometrically and refined using a riding model with C–H = 0.95 or 0.98 Å and *U*_iso_(H) = 1.2 or 1.5 *U*_eq_(C). A rotating-group model was applied for the methyl group.

### Crystal data

**Table:**

C_10_H_7_BrO_2_	*F*(000) = 944
*M**_r_* = 239.07	*D*_x_ = 1.809 Mg m^−^^3^
Orthorhombic, *P**b**c**a*	Mo *K*α radiation, λ = 0.71073 Å
Hall symbol: -P 2ac 2ab	Cell parameters from 9929 reflections
*a* = 10.8301 (2) Å	θ = 2.7–33.1°
*b* = 7.4630 (1) Å	µ = 4.64 mm^−^^1^
*c* = 21.7213 (3) Å	*T* = 100 K
*V* = 1755.62 (5) Å^3^	Block, colourless
*Z* = 8	0.26 × 0.19 × 0.18 mm

### Data collection

**Table:**

Bruker SMART APEXII DUO CCD diffractometer	3331 independent reflections
Radiation source: fine-focus sealed tube	2689 reflections with *I* > 2σ(*I*)
Graphite monochromator	*R*_int_ = 0.038
φ and ω scans	θ_max_ = 33.1°, θ_min_ = 1.9°
Absorption correction: multi-scan (*SADABS*; Bruker, 2009)	*h* = −16→16
*T*_min_ = 0.377, *T*_max_ = 0.482	*k* = −11→10
43601 measured reflections	*l* = −33→33

### Refinement

**Table:**

Refinement on *F*^2^	Primary atom site location: structure-invariant direct methods
Least-squares matrix: full	Secondary atom site location: difference Fourier map
*R*[*F*^2^ > 2σ(*F*^2^)] = 0.024	Hydrogen site location: inferred from neighbouring sites
*wR*(*F*^2^) = 0.058	H-atom parameters constrained
*S* = 1.04	*w* = 1/[σ^2^(*F*_o_^2^) + (0.0251*P*)^2^ + 1.0553*P*] where *P* = (*F*_o_^2^ + 2*F*_c_^2^)/3
3331 reflections	(Δ/σ)_max_ = 0.002
119 parameters	Δρ_max_ = 0.47 e Å^−^^3^
0 restraints	Δρ_min_ = −0.28 e Å^−^^3^

### Special details

**Table:**

Experimental. The crystal was placed in the cold stream of an Oxford Cryosystems Cobra open-flow nitrogen cryostat (Cosier & Glazer, 1986) operating at 100.0 (1) K.
Geometry. All esds (except the esd in the dihedral angle between two l.s. planes) are estimated using the full covariance matrix. The cell esds are taken into account individually in the estimation of esds in distances, angles and torsion angles; correlations between esds in cell parameters are only used when they are defined by crystal symmetry. An approximate (isotropic) treatment of cell esds is used for estimating esds involving l.s. planes.
Refinement. Refinement of F^2^ against ALL reflections. The weighted R-factor wR and goodness of fit S are based on F^2^, conventional R-factors R are based on F, with F set to zero for negative F^2^. The threshold expression of F^2^ > 2sigma(F^2^) is used only for calculating R-factors(gt) etc. and is not relevant to the choice of reflections for refinement. R-factors based on F^2^ are statistically about twice as large as those based on F, and R- factors based on ALL data will be even larger.

### Fractional atomic coordinates and isotropic or equivalent isotropic displacement parameters (Å^2^)

**Table:**

	*x*	*y*	*z*	*U*_iso_*/*U*_eq_	
Br1	0.176439 (12)	0.162250 (19)	0.544115 (6)	0.01904 (5)	
O1	0.36814 (9)	−0.10002 (14)	0.30247 (4)	0.01671 (18)	
O2	0.36732 (9)	−0.25959 (15)	0.18927 (5)	0.0228 (2)	
C1	0.33614 (11)	−0.03183 (18)	0.35866 (6)	0.0153 (2)	
C2	0.41792 (12)	0.03133 (19)	0.40293 (6)	0.0177 (3)	
H2A	0.5045	0.0339	0.3961	0.021*	
C3	0.36627 (13)	0.09040 (19)	0.45760 (6)	0.0176 (2)	
H3A	0.4182	0.1340	0.4895	0.021*	
C4	0.23784 (12)	0.08646 (19)	0.46632 (6)	0.0163 (2)	
C5	0.15642 (12)	0.02766 (19)	0.42185 (6)	0.0169 (2)	
H5A	0.0697	0.0287	0.4285	0.020*	
C6	0.20783 (12)	−0.03389 (18)	0.36629 (6)	0.0151 (2)	
C7	0.15886 (12)	−0.10978 (19)	0.31084 (6)	0.0165 (2)	
H7A	0.0743	−0.1294	0.3013	0.020*	
C8	0.25797 (12)	−0.14821 (19)	0.27452 (6)	0.0161 (2)	
C9	0.26702 (12)	−0.23368 (19)	0.21372 (6)	0.0171 (2)	
C10	0.14699 (13)	−0.2835 (2)	0.18369 (7)	0.0214 (3)	
H10D	0.1630	−0.3645	0.1491	0.032*	
H10A	0.1059	−0.1750	0.1686	0.032*	
H10B	0.0937	−0.3436	0.2138	0.032*	

### Atomic displacement parameters (Å^2^)

**Table:**

	*U*^11^	*U*^22^	*U*^33^	*U*^12^	*U*^13^	*U*^23^
Br1	0.02043 (7)	0.02101 (8)	0.01568 (7)	0.00053 (5)	0.00383 (5)	0.00000 (5)
O1	0.0123 (4)	0.0231 (5)	0.0147 (4)	−0.0003 (4)	0.0008 (3)	−0.0005 (4)
O2	0.0171 (4)	0.0314 (6)	0.0200 (5)	0.0005 (4)	0.0031 (4)	−0.0025 (4)
C1	0.0133 (5)	0.0173 (6)	0.0152 (5)	0.0004 (4)	0.0014 (4)	0.0025 (5)
C2	0.0133 (5)	0.0216 (7)	0.0180 (6)	0.0002 (5)	−0.0002 (5)	0.0007 (5)
C3	0.0157 (5)	0.0190 (6)	0.0183 (6)	0.0003 (5)	−0.0021 (5)	0.0011 (5)
C4	0.0179 (6)	0.0168 (6)	0.0141 (5)	0.0006 (5)	0.0029 (4)	0.0020 (5)
C5	0.0144 (6)	0.0182 (6)	0.0182 (6)	−0.0003 (5)	0.0019 (4)	0.0018 (5)
C6	0.0136 (5)	0.0168 (6)	0.0149 (5)	−0.0002 (5)	0.0008 (4)	0.0028 (5)
C7	0.0134 (5)	0.0192 (6)	0.0170 (6)	−0.0004 (5)	−0.0008 (4)	0.0022 (5)
C8	0.0132 (5)	0.0183 (6)	0.0167 (5)	−0.0005 (5)	−0.0008 (4)	0.0031 (5)
C9	0.0169 (6)	0.0178 (6)	0.0167 (6)	0.0002 (5)	−0.0003 (5)	0.0028 (5)
C10	0.0176 (6)	0.0277 (7)	0.0187 (6)	0.0001 (6)	−0.0028 (5)	−0.0001 (6)

### Geometric parameters (Å, º)

**Table:**

Br1—C4	1.9020 (13)	C5—C6	1.4062 (18)
O1—C1	1.3669 (16)	C5—H5A	0.9500
O1—C8	1.3862 (16)	C6—C7	1.4327 (19)
O2—C9	1.2245 (17)	C7—C8	1.3626 (18)
C1—C2	1.3898 (18)	C7—H7A	0.9500
C1—C6	1.3995 (18)	C8—C9	1.4699 (19)
C2—C3	1.3847 (19)	C9—C10	1.5013 (19)
C2—H2A	0.9500	C10—H10D	0.9800
C3—C4	1.4041 (19)	C10—H10A	0.9800
C3—H3A	0.9500	C10—H10B	0.9800
C4—C5	1.3795 (19)		
			
C1—O1—C8	105.63 (10)	C1—C6—C7	105.80 (11)
O1—C1—C2	125.63 (11)	C5—C6—C7	134.76 (12)
O1—C1—C6	110.70 (11)	C8—C7—C6	106.16 (11)
C2—C1—C6	123.67 (12)	C8—C7—H7A	126.9
C3—C2—C1	116.36 (12)	C6—C7—H7A	126.9
C3—C2—H2A	121.8	C7—C8—O1	111.71 (12)
C1—C2—H2A	121.8	C7—C8—C9	131.61 (12)
C2—C3—C4	120.61 (13)	O1—C8—C9	116.66 (11)
C2—C3—H3A	119.7	O2—C9—C8	121.15 (12)
C4—C3—H3A	119.7	O2—C9—C10	122.72 (13)
C5—C4—C3	123.05 (12)	C8—C9—C10	116.12 (12)
C5—C4—Br1	119.55 (10)	C9—C10—H10D	109.5
C3—C4—Br1	117.38 (10)	C9—C10—H10A	109.5
C4—C5—C6	116.86 (12)	H10D—C10—H10A	109.5
C4—C5—H5A	121.6	C9—C10—H10B	109.5
C6—C5—H5A	121.6	H10D—C10—H10B	109.5
C1—C6—C5	119.42 (12)	H10A—C10—H10B	109.5
			
C8—O1—C1—C2	179.08 (13)	C4—C5—C6—C1	−0.3 (2)
C8—O1—C1—C6	−0.81 (15)	C4—C5—C6—C7	177.41 (15)
O1—C1—C2—C3	−178.27 (13)	C1—C6—C7—C8	0.26 (15)
C6—C1—C2—C3	1.6 (2)	C5—C6—C7—C8	−177.71 (15)
C1—C2—C3—C4	−0.5 (2)	C6—C7—C8—O1	−0.78 (16)
C2—C3—C4—C5	−1.0 (2)	C6—C7—C8—C9	177.28 (14)
C2—C3—C4—Br1	177.76 (11)	C1—O1—C8—C7	1.00 (15)
C3—C4—C5—C6	1.4 (2)	C1—O1—C8—C9	−177.38 (12)
Br1—C4—C5—C6	−177.32 (10)	C7—C8—C9—O2	−178.33 (15)
O1—C1—C6—C5	178.69 (12)	O1—C8—C9—O2	−0.3 (2)
C2—C1—C6—C5	−1.2 (2)	C7—C8—C9—C10	2.6 (2)
O1—C1—C6—C7	0.35 (15)	O1—C8—C9—C10	−179.41 (12)
C2—C1—C6—C7	−179.53 (13)		

### Hydrogen-bond geometry (Å, º)

**Table:**

*D*—H···*A*	*D*—H	H···*A*	*D*···*A*	*D*—H···*A*
C7—H7*A*···O2^i^	0.95	2.45	3.3495 (16)	158

Symmetry code: (i) *x*−1/2, *y*, −*z*+1/2.
